# Personalized Dietary Advice to Increase Protein Intake in Older Adults Does Not Affect the Gut Microbiota, Appetite or Central Processing of Food Stimuli in Community-Dwelling Older Adults: A Six-Month Randomized Controlled Trial

**DOI:** 10.3390/nu15020332

**Published:** 2023-01-09

**Authors:** Kristina S. Fluitman, Madelief Wijdeveld, Mark Davids, Charlotte C. van Ruiten, Ilse Reinders, Hanneke A. H. Wijnhoven, Bart J. F. Keijser, Marjolein Visser, Max Nieuwdorp, Richard G. IJzerman

**Affiliations:** 1 Department of Internal Medicine, Amsterdam University Medical Center Location VUmc, 1081 HV Amsterdam, The Netherlands; 2 Amsterdam Public Health Research Institute, 1081 BT Amsterdam, The Netherlands; 3 Department of Vascular Medicine, Amsterdam University Medical Center, 1105 AZ Amsterdam, The Netherlands; 4 Department of Health Sciences, Faculty of Science, Vrije Universiteit Amsterdam and the Amsterdam Public Health Research Institute, 1081 HV Amsterdam, The Netherlands; 5 Nederlandse Organisatie voor Toegepast-Natuurwetenschappelijk Onderzoek (TNO) Earth, Life and Social Sciences, Department of Microbiology and Systems Biology, 3704 HE Zeist, The Netherlands; 6 Department of Preventive Dentistry, Academic Center for Dentistry Amsterdam, University of Amsterdam and VU University, 1012 WX Amsterdam, The Netherlands

**Keywords:** protein, appetite, gut microbiota, fMRI, older adults

## Abstract

Expert groups argue to raise the recommended daily allowance for protein in older adults from 0.8 to 1.2 g/kg/day to prevent undernutrition. However, protein is thought to increase satiety, possibly through effects on gut microbiota and central appetite regulation. If true, raising daily protein intake may work counterproductively. In a randomized controlled trial, we evaluated the effects of dietary advice aimed at increasing protein intake to 1.2 g/kg adjusted body weight/day (g/kg aBW/day) on appetite and gut microbiota in 90 community-dwelling older adults with habitual protein intake <1.0 g/kg aBW/day (N_intervention_ = 47, N_control_ = 43). Food intake was determined by 24-h dietary recalls and gut microbiota by 16S rRNA sequencing. Functional magnetic resonance imaging (fMRI) scans were performed in a subgroup of 48 participants to evaluate central nervous system responses to food-related stimuli. Both groups had mean baseline protein intake of 0.8 ± 0.2 g/kg aBW/day. At 6 months’ follow-up this increased to 1.2 ± 0.2 g/kg aBW/day for the intervention group and 0.9 ± 0.2 g/kg aBW/day for the control group. Microbiota composition was not affected, nor were appetite or brain activity in response to food-related stimuli. Increasing protein intake in older adults to 1.2 g/kg aBW/day does not negatively impact the gut microbiota or suppress appetite.

## 1. Introduction

Undernutrition is an important problem in community-dwelling older adults, and associated with higher risk of age-related disease and functional impairment. In Europe, the prevalence of undernutrition ranges from 7–16% in community-dwelling older adults to 18–33% in institutionalized older adults [1,2]. Sufficient protein intake in older adults is needed to maintain muscle mass and functionality, and to prevent undernutrition. Furthermore, ageing adults undergo various physiological changes, resulting in increased protein requirements [3,4]. That is why expert groups argue that the current recommended daily allowance (RDA) of protein for older adults should be raised from 0.8 g/kg/day [5] to 1.2 g/kg/day [3,6]. Currently, protein supplementation is already an effective and widely accepted strategy to treat (imminent) undernutrition in older adults [3,7]. However, protein is also considered the most satiating of macronutrients [8] and elevated protein intake has even been suggested as a means to lose weight for people with obesity [9]. Indeed, in the general population, increasing dietary protein leads to reduced energy intake [10]. However, specific effects in older adults are hitherto largely unknown [11]. If increasing dietary protein does lead to reduced appetite in older adults, increasing the RDA of protein for this population could work counterproductively in the battle against undernutrition, especially since poor appetite has been found to be the most important risk factor for undernutrition in older adults [12].

Protein intake may influence appetite in several ways. It is thought to delay gastric emptying [13] and alter appetite regulating (gut) hormones [8]. Furthermore, as measured with functional magnetic resonance imaging (fMRI) scans, increased protein intake in overweight or obese individuals has been associated with lower central nervous system (CNS) responses in brain reward and satiety areas when viewing food cues [14,15,16], suggesting a direct effect on central appetite regulation. Possibly, dietary protein exerts its satietogenic effect partly through the gut microbiota as well [17]. Through the fermentation of otherwise indigestible proteins and polysaccharides, the gut microbiota produce metabolites such as short-chain fatty acids (SCFA) with endocrine and paracrine properties, affecting appetite through the gut–brain axis [18]. In humans, increasing protein intake has tended to decrease SCFA-producing bacteria, including various *Ruminococcus* subspecies [19]. To our knowledge, the effects of increasing dietary protein intake to the suggested 1.2 g/kg/day on appetite, the CNS responses to food stimuli, and the gut microbial composition have not been investigated in older adults.

The aim of this study was to evaluate the effects of dietary advice aimed at increasing protein intake on appetite and gut microbial composition in a subgroup of 90 participants from the PROMISS trial. This concerns an ancillary study addressing the secondary outcomes of a large, 6-month, multicenter randomized controlled trial implementing dietary advice aimed at increasing protein intake to the suggested 1.2 g/kg adjusted body weight/day (kg aBW/day) in older adults with habitual low protein intake (<1.0 g/kg aBW/day) [20,21]. Appetite was assessed with a standardized questionnaire and visual analogue scores (VAS). The gut microbiota were assessed with 16S rRNA sequencing. Moreover, we performed fMRI scans measuring responses in reward and satiety areas of the brain to food cues. We hypothesized that protein will suppress appetite, modulate brain activity in response to food cues, and significantly alter the gut microbial composition, specifically decreasing SCFA-producing species.

## 2. Materials and Methods

This study concerns the ancillary outcome of the 6-month, multicenter, randomized controlled Prevention Of Malnutrition In Senior Subjects in the EU (PROMISS) trial, performed from 2018 to 2020 at two study sites: the University of Helsinki, Helsinki, Finland and the Vrije Universiteit Amsterdam, Amsterdam, the Netherlands [20]. PROMISS’s primary objective was to examine the (cost-)effectiveness of personalized dietary advice aimed at increasing protein intake to 1.2 g/kg aBW/day during a 6-month period on improving physical functioning in community-dwelling older adults with habitual protein intake <1.0 g/kg aBW/day [21]. The full protocol of the PROMISS trial has been published elsewhere [20]. In this current study, we evaluate the effect of the intervention on subjective appetite, CNS responses to food cues and the gut microbiota composition. Written informed consent was obtained from all participants prior to study enrollment. The PROMISS trial was registered at ClinicalTrials.gov (NCT03712306) and was approved by both the Institutional Review Boards of the Amsterdam UMC, location VUMC in Amsterdam, the Netherlands (approval code: 2018.399), and by the University of Helsinki (approval code: HUS/1530/2018). It was conducted in accordance with the Declaration of Helsinki (version 2013).

### 2.1. Participants

In the PROMISS trial, 276 participants were included. PROMISS baseline inclusion criteria were: age ≥ 65 years, community-dwelling, habitual protein intake < 1.0 g/kg aBW/day determined by three food-diary-assisted 24-h dietary recalls, body mass index (BMI) ≥ 18.5 kg/m^2^ and ≤32.0 kg/m^2^, and ability to walk 400 m within 15 min without the use of a walker and no rest > 60 s [21]. Exclusion criteria were: adherence to a vegan diet, severe food allergies, purposefully lost or gained >3 kg in past 3 months, diagnosed severe kidney disease, diagnosed type 1 diabetes or insulin dependent type 2 diabetes, diagnosed eating disorder, severe acute heart disease in the past 3 months, and poor cognitive status determined by a Mini-Mental State Examination score ≤ 20 [22]. Additional baseline exclusion criteria for participation in this microbiota ancillary study were: diagnosed inflammatory bowel disease, prolonged institutionalization (>4 weeks) in the past 3 months, and use of systemic antibiotics in the past 3 months. Furthermore, 48 willing participants from the microbiota ancillary study (27 in the diet intervention group, 21 in the control group) who did not have diagnosed mental disorders or contraindications for MRI scans, underwent functional MRI (fMRI) scans at baseline and 6-month follow-up to evaluate the CNS responses to food-related stimuli. Since random assignment to treatment arms was only performed for the overall PROMISS trial, it could not be ensured that stratification for sex and baseline habitual protein was fully implemented in this ancillary trial. Therefore, baseline characteristics were statistically tested to assess between-group differences.

**Figure 1:**
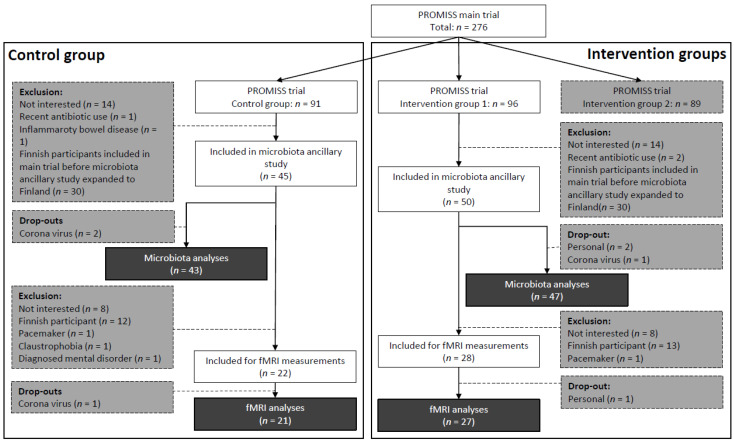
Flowchart for inclusion of participants in the microbiota ancillary study and fMRI measurements of the PROMISS trial. Darkest grey is final number of included participants per group, per substudy. Grey is the number of exclusions. fMRI: functional magnetic resonance imaging; PROMISS: Prevention Of Malnutrition In Senior Subjects in the EU.

As described previously [20], PROMISS trial participants were randomized into 3 groups: intervention group 1 with dietary advice aimed at increasing protein intake (*n* = 96), intervention group 2 with dietary advice aimed at increasing protein intake as well as consuming protein in close proximity to physical activity (*n* = 89), and a control group (*n* = 91). Participants were allocated in a 1:1:1 ratio, block size of 3, and stratified on sex and baseline habitual protein intake (<0.9 or 0.9–1.0 g/kg aBW/day) to ensure homogeneous distribution of participants across groups. Only participants from intervention group 1 (diet group) or the control group were eligible for this ancillary microbiota study. Initially, only participants from the Dutch PROMISS site were recruited. However, recruitment was later expanded to the Finnish study site as well, due to slow inclusion rate. Finnish participants were not eligible for the fMRI measurements due to logistic reasons. In total, 95 PROMISS participants were included in the ancillary microbiota study, of whom 5 dropped out. Fifty of these participants also had fMRI scans made, of whom 2 dropped out (Figure 1).

### 2.2. Intervention

All participants were invited to the clinic for assessments at baseline (prior to randomization), 3 month, and 6 month follow-up. The dietary advice was given orally and in writing. It was aimed at increasing protein intake to 1.2 g/kg aBW/day with at least one meal containing ≥35 g protein, while not increasing total daily energy intake. The advice was personalized based on habitual dietary characteristics, measured body weight, and personal preferences regarding food consumption and preparation [20]. Complete dietary adjustments and protein-enriched food sources in the Dutch arm of the PROMISS study are described elsewhere [23]. Here is established that most increased protein sources at 6-month follow-up consisted of animal-based products (11.0 g protein/day compared to 2.1 g protein/day of plant-based products) [23]. For those with a BMI of 18.5–22.0 kg/m^2^, or 25.0–32.0 kg/m^2^ (age ≤ 70), or a BMI of 27.0–32.0 kg/m^2^ (age > 70) actual body weights were adjusted to the nearest weight that would place an individual in the healthy BMI range. This prevents underestimation or overestimation of protein requirements due to increased protein needs or excessive adipose tissue. Prior to each visit, dietary intake was measured by 3 consecutive food-diary-assisted 24-h dietary recalls.

### 2.3. Self-Reported Appetite

Participants filled out the 4-item Simplified Nutritional Appetite Questionnaire (SNAQ) at baseline and 6-month follow-up, its score ranging from 4 (poor appetite) to 20 (good appetite) [24]. The last question was changed in translation from English to Dutch and Finnish from the number of meals eaten per day to the number of meals and/or snacks eaten per day. Answering categories were: less than 4 times a day; 4 times a day; 5 times a day or more than 5 times a day. Participants who took part in the fMRI measurements also filled out a VAS scale consisting of 6 appetite-related questions (score: 0–10) to report on hunger and satiety immediately after each fMRI scan.

### 2.4. Biosampling and 16S rRNA Sequencing

Participants were asked to collect a fecal sample at home at baseline and at the six month follow-up visit, in a sterile container (Sarstedt, Nümbrecht, Germany). Upon collection participants immediately stored the fecal sample in their −20 °C freezer until transfer to the research center in a provided icepack. Samples there were kept in a −20 °C freezer until transfer to central storage at −80 °C once a week. Time of sample production, storage at −20 °C, and storage at −80 °C was noted. 

After all stool samples were collected, they were shipped to the Amsterdam University Medical Center, Amsterdam, the Netherlands on dry ice for DNA extraction. Total genomic DNA was isolated using an adapted repeated bead-beating method [25]. Extracted DNA was then shipped to the Wallenberg Laboratory (Sahlgrenska Academy at University of Gothenburg, Gothenburg, Sweden). There, the composition of fecal microbiota was profiled by sequencing the V4 region of the 16S rRNA gene on a MiSeq system (RTA version 1.17.28, bundled with MCS version 2.5; Illumina, San Diego, CA, USA) with 515F and 806R primers designed for dual indexing [26] and the V2 kit (2 × 250 bp paired-end reads; Illumina, San Diego, CA, USA). The 16S rRNA genes from each sample were amplified in volumes of 25 μL containing 1 × 5 PRIME HotMasterMix; 5 PRIME, Hotmaster Inc., Manchester, UK), 200 nM of each primer, 0.4 mg/mL bovine serum albumin (BSA), 5% dimethylsulfoxide, and 20 ng of genomic DNA. Polymerase Chain Reaction (PCR) was carried out under the following conditions: initial denaturation for 3 min at 94 °C; followed by 25 cycles of denaturation for 45 s at 94 °C; annealing for 60 s at 52 °C; elongation for 90 s at 72 °C; and a final elongation step for 10 min at 72 °C. PCR products were purified with the NucleoSpin Gel and PCR Clean-Up kit (Macherey-Nagel, Dueren, Germany), and quantified using the Quant-iT PicoGreen dsDNA kit (Invitrogen, Waltham, MA, USA). Purified PCR products were diluted to 10 ng/μL and pooled in equal amounts. The pooled amplicons were purified again using Ampure magnetic purification beads (Agencourt, Beverly, CA, USA) to remove short amplification products. Positive and negative DNA extraction controls, as well as positive PCR controls, were included in analysis. Amplicon reads were merged and processed using USEARCH [27]. Merged reads with expected error rates higher than 1 were filtered after which amplicon sequence variants (ASVs) were inferred using UNOISE [28]. The unfiltered reads were used to determine the ASV abundances. Taxonomy was assigned using the RDP classifier [29] and SILVA [30] 16S ribosomal database V132. ASV sequences were aligned using DECIPHER (2.18.1) and a phylogenetic tree was generated by optimized neighbor-joining tree estimation using phangorn (2.7.0) (CRAN.R-project; https://CRAN.R-project.org/package=phangorn (accessed on 28 November 2022)).

### 2.5. MRI Scanning

MRI scans were made after an overnight fast of at least 12 h, except for water (coffee or tea also not allowed). Three hours prior to the MRI scan, all participants were instructed to consume a single standardized sandwich in order to normalize food intake between subjects on the day of testing. Due to travel restrictions during the COVID pandemic, 14 participants (control: *n* = 5, diet: *n* = 9) could not visit the research facility in time for their 6-month follow-up scan. These visits were postponed by several weeks (median days to follow-up (interquartile range (IQR)) was 182 (179–203) and 186 (182–199) for diet group and control group, respectively). Participants from the diet groups whose follow-up MRI scans were postponed due to COVID continued their diets until the follow-up MRI scan could be made. MRI acquisition and analyses have been described in detail previously [31]. In brief, MRI data were acquired on a 3.0 tesla Signa HDxt scanner (General Electric, Milwaukee, WI, USA) using a 32-channel receive-only head coil. Structural brain images were obtained using a T1-weighted sequence. fMRI data were acquired using an echo-planar-imaging T2* blood-oxygenation-level-dependent pulse sequence (repetition time 2160 ms, echo time 30 ms, matrix 64 × 64, 211 mm^2^ field of view, flip angle 80° with 40 ascending slices per volume (3 mm thickness, 0 mm gap), which provided whole-brain coverage. Preprocessing was performed using FMRIPREP v20.0.6 (Poldrack lab, Center for Reproducible Neuroscience, Stanford University, Stanford, CA, USA) [32,33] (RRID: SCR_016 216). Each T1-weighted (T1w) scan was normalized to Montreal Neurologic Institution (MNI) space [34] (TemplateFlow ID: MNI152NLin2009cAsym). Functional data preprocessing included motion correction (FLIRT) [35] and distortion correction (3dQwarp) [36], followed by co-registration to the T1w image using FLIRT (FSL 5.0.9.) (Oxford Centre for Functional Magnetic Resonance Imaging of the Brain, Oxford, UK) [37]. Independent-component-analysis-based automatic removal of motion artifacts (ICA-AROMA) was used to non-aggressively denoise the data [38]. Hereafter, data were spatially smoothed (6 mm full-width at half-maximum (FWHM)) and high-pass filtered (100 s) within FSL/FEAT (FSL/FEAT v6.0.0) (created by the Analysis Group, Oxford University, Oxford, UK).

### 2.6. Functional MRI Experimental Design

Participants were submitted to two separate fMRI tasks: they were presented with pictures of food and non-food (picture task), and with actual palatable food receipt (palatable food task). The fMRI picture task was performed as described previously [31]. In short, this fMRI task consisted of a 10-minute presentation of pictures selected from the following three categories: (1) high-calorie food items, (2) low-calorie food items, and (3) non-food items. The pictures were presented via the software E-Prime version 1.2 (Psychology Software Tools, Inc., Pittsburgh, PA, USA) in a block design. Each fMRI picture task consisted of 3 runs, each comprising six blocks of 21 s (7 pictures, 2.5 s each with a 0.5 s inter-stimuli interval of a blank screen). Within each run, two blocks of each category were presented. The blocks were separated with a 9 s black screen with a fixation cross. The order of the categories was randomized per run and per session. Pictures were matched for type, shape, and color.

Each palatable food task consisted of 64 trials in total as described previously [39]. Chocolate milk (Chocomel (FrieslandCampina, Amersfoort, Netherlands); 73 kcal, 1.6 g fat, 11 g sugar per 100 mL) was administered as a palatable food stimulus. A tasteless solution was used as a control stimulus, designed to mimic the natural taste of saliva (consisting of 2.5 mM sodium bicarbonate (NaHCO_3_) and 25 mM potassium chloride (KCl)) [40]. During each trial a visual cue was presented: either an orange triangle or a blue star that signaled either the delivery of 0.4 mL of chocolate milk or tasteless solution, respectively. Images were presented for 2 s in random order, followed by a 3 s blank screen with a fixation cross and 2 s of stimulus delivery. Participants were instructed to keep the solution in their mouths for 5 s until the ‘swallow’ cue appeared. The next trial was started after 1–7 s. In 40% of the trials, however, the cue was not followed by a stimulus delivery in order to maintain an unconditioned response to the receipt of the solutions. Participants were unaware whether a presented image would be paired or unpaired with the delivery of chocolate milk or a tasteless solution. The order of trials was randomized. The picture food task and palatable food task scan comprised 277 and 426 volumes, respectively.

### 2.7. Statistics

All univariate data were analyzed by SPSS software version 22 (SPSS Inc., Chicago IL, USA). Data are depicted as mean ± standard deviation, median and interquartile ranges, and number and percentages. Between-group baseline differences were tested with independent Student’s *t*-tests, Mann–Whitney U tests, and Fisher’s exact tests depending on Gaussian distribution. Linear regression analyses adjusted for baseline values were run to test the effect of the dietary advice on between-group differences in SNAQ scores and VAS appetite scores. Because dietary data were collected longitudinally (at 3- and 6-month follow-up), mixed-effects models were run to test the effect of the dietary advice on macronutrient intake. First, time (visit), group (intervention versus control) and baseline value were added to the model as fixed effects to each model. A random intercept was added to the models to take into account the dependency of the repeated observations among the participants. For any model where macronutrient intake was significantly different between groups over time, separate measurements were done for 3 and 6 month follow-up, adjusting for baseline values. For all statistical tests, the 2-sided significance threshold was set to a *p*-value of 0.05. Linear regression analyses adjusted for baseline values were run to test between-group differences in fMRI blood-oxygen level dependent (BOLD) signal differences in pre-defined brain regions of interest (ROIs). Previous dietary protein intervention studies have detected significant differences in microbiota composition between groups of 20–47 subjects [41,42]. Moreover, based on the results of previous task-based fMRI studies on appetite [43,44], it was estimated that 24 participants per group rendered sufficient power. We therefore deduced our sample size to be sufficient. However, a formal power calculation was not conducted since our study does not concern the primary outcome of the trial. Due to the exploratory nature of our analyses, we did not apply *p*-value correction for multiple comparisons.

fMRI processing and analyzing was performed using FSL version 5.0.9 (created by the Analysis Group, Oxford University, Oxford, UK). First, four contrasts were calculated, two for each task [43,45]. For the picture task, BOLD signal intensity in response to non-food images was subtracted from BOLD signal intensity in response to all food images (food vs. non-food, contrast 1), and from high-calorie food images (high caloric vs. non-food, contrast 2). For the palatable food task, the BOLD signal in response to the anticipation of a tasteless solution was subtracted from BOLD signal in response to the anticipation of chocolate milk (anticipation chocolate milk vs. anticipation tasteless solution, contrast 3). Similarly, brain activity in response to receiving a tasteless solution was subtracted from brain activity in response to receiving chocolate milk (receipt chocolate milk vs. receipt tasteless solution, contrast 4). These contrasts are an approximation for a person’s response to visual food stimuli, inducing a food craving sensation in semi-fasted state, and to anticipating and receiving palatable food (i.e., chocolate milk), the latter inducing a central reward response. First, these contrasts were explored on a whole-brain level, considering all regions of the brain. Peaks of activity on whole-brain level were examined using the FEAT function of FSL 5.0.6 (FMRIB Software Library; University of Oxford, Oxford, UK) [46]. Whole-brain analyses consisted of main-effects analysis and group-effects analysis. Main effect (one-sample *t*-test) was assessed in order to validate the effectiveness of each task among all subjects, regardless of intervention. Results were considered significant at *p* > 0.05, on cluster extent, maintaining a Z threshold of 3.1, corrected for multiple comparisons using family-wise error (FWE). Cluster information was obtained using the cluster tool from FSL. For the main effects, brain activity in response to each experimental condition was compared to the contrasting condition among all participants at baseline to verify that the contrasting experimental conditions did elicit significant differential responses in brain activity. For the group effects, the four contrasts were compared between the diet and control groups at follow-up. For the main-effect analyses the contrasts of all participants were entered into a one-sample *t*-test in FSL and a statistical map was created for each contrast. For the group-effect analyses whole-brain activation was compared at voxel level with paired *t*-tests, FWE corrected for multiple comparisons.

After the whole-brain analysis, between-group differences for all four contrasts were specifically analyzed for four pre-defined anatomical ROIs: the amygdala, caudate nucleus, putamen, and insula. As part of the basal ganglia, the caudate and putamen are implicated in reward processing and conditioning and have previously been found to play a complex integrating role in food-related reward signals to behavior [47]. The amygdala is the primary brain region regulating appetite and assigning emotional content to food-related stimuli [48]. The insula is part of a neural circuit involved in the preventing of overeating and terminating feeding upon satiation [49]. Furthermore, all ROIs are related to food reward and motivation, based on previous studies [40,50]. Anatomical masks containing unilateral ROIs were used to perform ROI analyses. The masks were created with fslmaths using the Harvard–Oxford cortical (insula mask) and subcortical (amygdala, caudate, and putamen mask) atlases and thresholds at 60%. Consequently, for each contrast, the mean BOLD signal intensity of every single participant was extracted per anatomical region.

All microbiota-related analyses were performed in R (version 4.0.0) [51]. Specifically the Phyloseq package [52], Vegan package [53], and ggplot2 package [54] were used for analysis and visualization; 16S data were rarified without replacement to 30,000 counts. First, 3 alpha-diversity metrics were calculated (i.e., metrics to determine within-sample diversity): observed taxa, Shannon diversity index, and Faiths Phylogenetic Diversity (FPD). Linear mixed-model analysis was used to test for intervention-induced changes in these alpha-diversity metrics. In these models an interaction term for intervention × time and a random intercept for participant were included. Next, permutational multivariate analysis of variance (PERMANOVA) was used to test compositional differences induced by the intervention, based on both Bray–Curtis dissimilarity and weighted UniFrac distances. In addition, multilevel principal component analysis (PCA) was used on centered log-ratio (CLR)-transformed counts to view compositional shifts. To check for heterogeneous effect of the intervention, within-subject distances were compared between groups by means of dispersion analysis. Univariate regression on the microbiome count data was performed using linear mixed models from the DESeq2 package (Bioconductor Project, University of North Carolina, Chapel Hill, NC, USA), version 1.20.0 and the dream package, version 1.23.0 (Institute for Statistics and Mathematics, Wirtschaftsuniversität, Vienna, Austria) with the variancePartition extension [55,56,57]. This combination of packages provides functions for differential abundance testing using negative binomial linear mixed models for repeated measures data. Benjamini–Hochberg correction for multiple testing was applied [58]. A *p*-value < 0.05 was considered statistically significant.

## 3. Results

### 3.1. Participants

Overall, no differences were found between the intervention and control groups in baseline characteristics, including age, sex, BMI, Mini-Mental State Exam (MMSE) score, and level of education. This was true for both our ancillary microbiota study and the subsample that took part in fMRI measurements. All baseline characteristics are depicted in Table 1.

### 3.2. Dietary Intake

The participants that took part in this ancillary study did not differ based on energy and macronutrient intake at baseline (Table 1). On average, participants from the diet group increased their protein intake from mean 0.8 g/kg aBW/day at baseline to mean 1.3 g/kg aBW/day at 3 months and to mean 1.2 g/kg aBW/day at 6 months. In contrast, the control group had a protein intake of 0.9 g/kg aBW/day at both 3 and 6 months (*p* < 0.0001) (Table 2). This difference in protein intake was found statistically significant based on linear mixed models corrected for baseline measurement, which was confirmed in linear regression models corrected for baseline for each time point for g/kg aBW/day (B = 0.4, *p* < 0.001; B = 0.3, *p* < 0.001, for 3 and 6 month follow-up, respectively) and for g/day (B = 30.1, *p* < 0.001; B = 26.2, *p* < 0.001, for 3 and 6 month follow-up, respectively). Daily energy and carbohydrate intake were also significantly affected by the intervention based on linear mixed models (*p* = 0.0008 and 0.0367, respectively); however, when performing comparisons between groups for each time point separately, using linear regression models, no significant differences were found. Fat intake was not significantly affected by the intervention. Daily energy, carbohydrate and fat intake were not significantly affected by the intervention for the subsample of participants that took part in the fMRI measurements.

### 3.3. Appetite Assessments

There were no baseline differences in SNAQ scores in the total microbiota subsample or in the subsample of participants that took part in the fMRI measurements between the diet group and the control group (*p* = 0.965 and *p* = 0.978, respectively), nor were there baseline differences in average VAS appetite scores (4.7/10 in the diet group and 5.3/10 in the control group, *p* = 0.104) (Table 1). The intervention did not affect self-reported appetite, since SNAQ scores and VAS scores did not differ between the diet and control groups at follow-up (Table 3).

### 3.4. fMRI Assessments

Table 4 presents all brain areas that showed significant (*p* < 0.05 FWE whole brain corrected) differential activation across all participants at baseline between contrasting conditions (the results of the whole-brain main-effects analyses). As can be seen several areas, including the left insula, respond differently to watching food pictures or high-caloric food pictures versus non-food pictures. However, no significant differences in brain activation could be found between the contrasting conditions of the palatable food task. Moreover, there were no significant differences on whole-brain level between the diet and control groups at follow-up for either of the tasks.

The ROI analyses for the 6-month follow-up visit showed there was an increase in signal in the right insula in response to anticipating chocolate milk in the intervention group (B = 0.07, *p* = 0.045); therefore, a difference of 0.07 percentage point between the intervention and control groups was observed. No further altered activity in the diet group compared to the control group was found for any of the contrasts, in any of the ROIs (Table 5).

### 3.5. Gut Microbiota

**Figure 2:**
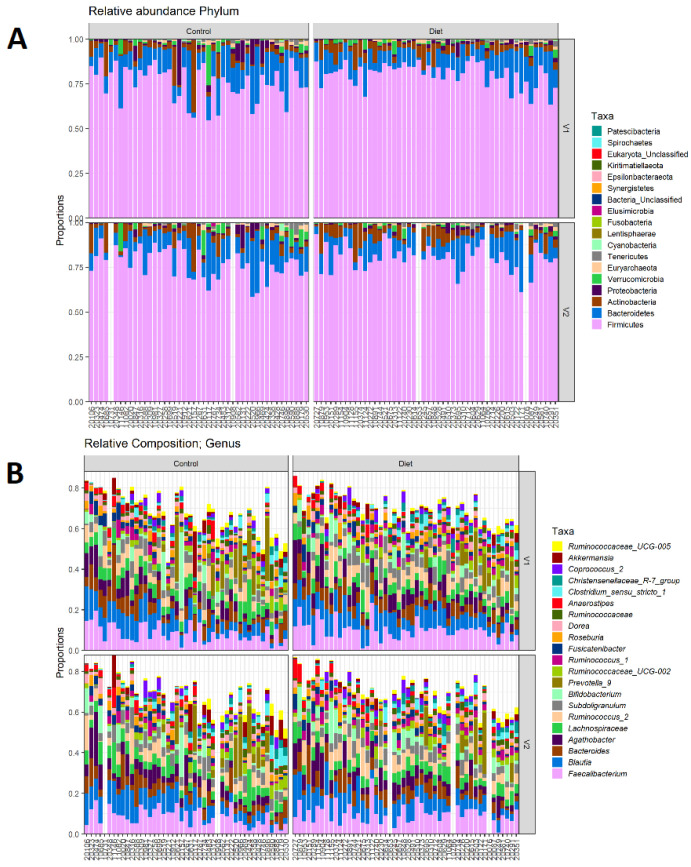
Microbiota composition for each participant. (**A**) Relative abundance of bacterial phyla for each participant, stratified to intervention groups and visits (V1: baseline visit, V2: follow-up visit). *Firmicutes* are the most abundant, followed by *Bacteroidetes*, *Actinobacteria*, and *Proteobacteria*. (**B**) Same as A, but for 20 most abundant bacterial genera. There are no differences in phylum abundance or genus abundance between groups.

**Figure 3:**
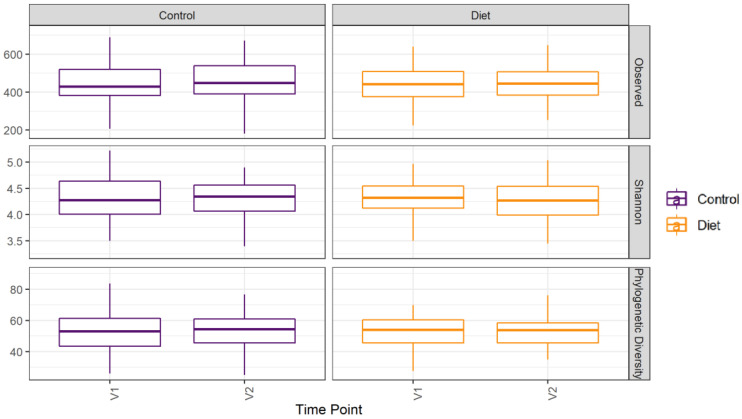
Microbiota alpha diversity was not affected by increasing protein intake. Alpha diversity for the control and diet groups at baseline (V1) and 6 month follow-up (V2) based on observed number of taxa, Shannon index, and Faith’s Phylogenetic Diversity index. Observed taxa is the total number of unique ASVs observed in a sample; it is a metric for richness. Shannon is an evenness diversity metric: a more even distribution results in a higher diversity. FPD takes into account the phylogeny of the observed taxa; it is a metric for genetic diversity. Linear mixed models were used to test for intervention-induced changes in alpha diversity. In these models an interaction term for intervention × time and a random intercept for participant were included. Dietary advice aimed at increasing protein intake did not affect any of the alpha-diversity measures. ASV: amplicon sequence variants; FPD: Faiths Phylogenetic Diversity.

**Figure 4:**
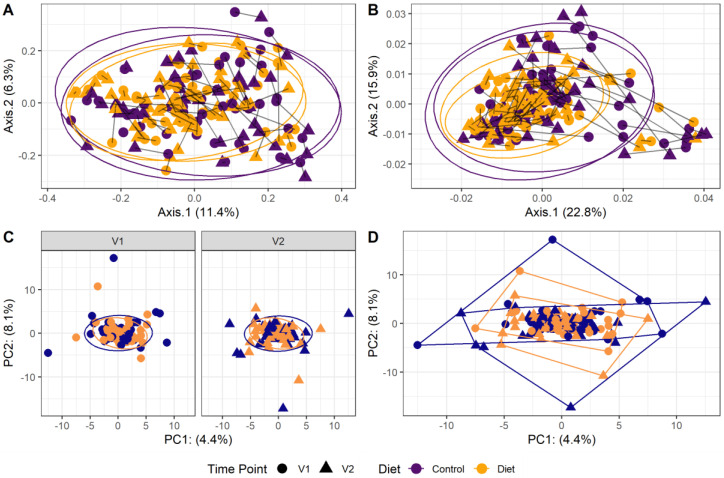
Microbiota beta diversity was not affected by increasing protein intake. (**A**,**B**): Principle coordinate analysis (PCoA) based on Bray–Curtis dissimilarity (**A**) and weighted UniFrac (**B**) measures. These are measures used to evaluate overall microbial compositional differences between groups. Each point indicates one sample from one participant (yellow: diet group, purple: control group, dot: baseline visit (V1), triangle: 6 month follow-up visit (V2)). Points from one participant are linked. The closer the points are mapped together, the more the microbial composition is alike. PERMANOVA analysis showed that overall microbiota composition based on the Bray–Curtis dissimilarity measure or on weighted UniFrac did not change significantly from baseline to follow-up in the intervention compared to the control group. (**C**,**D**): Multilevel principal component analysis (PCA) stratified for baseline (V1) and 6 month follow-up visits (V2) (**C**) and for all visits combined (**D**). There was no significant (*p* = 0.43) effect of the dietary intervention on this beta-diversity measure.

The microbiota composition for each participant, stratified per group and visit, are depicted in Figure 2. None of the three alpha-diversity metrics (i.e., within-sample diversity) calculated were affected by the dietary intervention (Figure 3). PERMANOVA analysis showed that overall microbiota composition based on the Bray–Curtis dissimilarity measure or on weighted UniFrac did not change significantly from baseline to follow-up in the intervention compared to the control group (Figure 4A,B). Multilevel PCA of the CLR transformed data also showed no significant (*p* = 0.43) effect of the dietary intervention (Figure 4C,D). Specific microbial species count, assessed by univariate regression using the DESeq2 package, also did not differ between the groups. Since different protein sources might elicit different effects, we also performed a dispersion analysis which showed that the intervention did not perturb the microbiome compared to the control (*p* = 0.31). Moreover, specific microbial species count was also not affected by the intervention using a negative binomial linear mixed-effect model from the DESeq2 package.

## 4. Discussion

Our study demonstrates that dietary advice aimed at increasing protein intake from an average of 0.8 g/kg aBW/day to 1.2 g/kg aBW/day in community-dwelling older adults did lead to an increase in protein intake, but does not affect gut microbiota composition, appetite, or brain activity in response to food-related stimuli. As the PROMISS study has shown that the intervention does improve 400 m walk time or leg strength [21], it is valid to conclude that the recommended increase in RDA for protein to 1.2 g/kg aBW/day can be a safe strategy to improve physical function, with no adverse effects on appetite or microbiota composition.

In our study we measured the effect of a dietary intervention on appetite using an adapted version of the SNAQ appetite questionnaire [24]. Moreover, the 48 participants who underwent fMRI scans were also asked to rate their appetite using 6 VAS scales. Neither the SNAQ appetite scores nor the VAS appetite ratings were affected by our dietary intervention. In addition to subjective ratings of appetite, we also examined the effects of increasing protein intake on fMRI-measured brain responses to food cues. These food cues were provided in the form of food versus non-food images or the anticipation or receipt of chocolate milk versus a tasteless solution, which are considered potent activators of the brain’s reward system [59]. By comparing the changes in CNS signaling between participants from our diet and control groups, we aimed to evaluate whether there is a neural basis for possible protein-induced effects on appetite in older adults. Although several studies have demonstrated such differential brain activation patterns in adults with obesity [60] or weight gain [61], we are the first to study the effect of increasing protein intake on fMRI-measured brain activation in older adults. In line with our findings with regard to self-rated appetite, we found no significant alterations in brain responses in the protein intervention group that could lead to a decrease in energy intake. We did find an increase in signal in the right insula in response to anticipating chocolate milk in the protein intervention group (*p* = 0.045), indicating a slight increase in appetite, rather than the apprehended decrease. Our findings contradict previous studies in younger adults with obesity, in whom protein intake did alter CNS control of appetite [14,15,16]. Possibly, older adults experience a blunted response to the satietogenic properties of dietary protein [62]. It has been shown that, upon a dietary protein intervention, older men had delayed gastric emptying compared to younger men, but did not report decreased appetite (measured by VAS scores), and experienced less suppression of energy intake during a consecutive ad libitum meal [62]. Altogether, our current results indicate that protein supplementation to 1.2 g/kg aBW/day is a safe strategy to improve physical function [21] without any adverse effects on appetite in older adults.

We hypothesized that protein-induced changes in self-rated appetite or brain activity patterns would be partially mediated by the gut microbiota through the gut–brain axis. However, in addition to not affecting appetite or CNS responses to food cues, our intervention did not affect the composition of the gut microbiota. Neither the alpha diversity (within-sample diversity), nor the beta diversity (between-samples dissimilarity), nor the bacterial abundance of individual taxa were affected. This finding contradicts the study by David et al. [63], which demonstrated that a purely animal-based diet (high in protein and fat, low in fiber) in young adults altered the microbiota composition one day after the animal diet reached the gut [63]. Normally, most protein is digested in the small intestine and only excess protein reaches the colon where most of the gut microbiota resides [64]. Possibly the increase of protein intake in our study was not drastic enough to effectuate a similar colonic increase in protein. To illustrate: David et al. [63] increased protein intake almost 100%, whereas we altered it by about 50%. Furthermore, the use of diverse protein sources in our study, including both animal and plant-based protein, might have prevented a uniform microbial effect. Since increased proteolytic (compared to saccharolytic) fermentation increases the production of detrimental metabolites with cytotoxic and carcinogenic attributes, increases luminal pH, and favors pathogen proliferation [64], the lack of protein-induced changes to the microbiota in our study can be considered a positive finding. 

### Strengths and Limitations

An important strength of this study was that the dietary advice given to participants was personally tailored by nutritionists to each participant’s personal eating behaviors and preferences to increase adherence. Moreover, the advice was adjusted during the study period based on the participants’ feedback to further improve compliance. Based on the 24-h recalls, the advice did succeed in raising the average protein intake from 0.8 g/kg aBW/day to 1.2 g/kg aBW/day. Although the increase in protein intake was moderate, it was in line with the recommended raise in RDA for protein intake in older adults suggested by several expert groups [3,65]. It is therefore an accurate depiction of the health effects that can be expected if the RDA for protein intake in older adults would increase to 1.2 g/kg aBW/day. Moreover, the increased protein intake was sufficient to increase 400 m walk time and leg strength, as demonstrated by the PROMISS trial itself [21]. Nevertheless, some limitations are to be acknowledged. First, this study describes the results of a trial that was primarily designed to study the effect of increasing protein intake on physical functioning [20]. The effects on appetite and the gut microbiota were secondary outcomes studied in a subpopulation, therefore this study should be considered exploratory and hypothesis-generating. However, it is still a large population compared to other dietary protein interventions [66]. Second, although compliance with the diet was measured by 24-h recalls, no urinary samples were obtained to objectively assess protein intake. Third, not every participant from the intervention groups reached the recommended dietary protein intake of >1.2 g/kg aBW/day. This was obtained for a majority of the intervention group (53%), however. Nevertheless, the advisory or coaching method for a dietary intervention is more representative of the way that dietary interventions are generally implemented by clinical dieticians [67]. Fourth, it has to be noted that isocaloric dietary interventions increasing one particular macronutrient always result in the relative decrease of other macronutrients. Finally, due to the exploratory nature of this study, the effects of the protein intervention on metabolic markers such as muscle mass or insulin resistance was not assessed. We also did not perform measurements of postprandial leptin or ghrelin, or plasma metabolomics, transcriptomics, or fecal short-chain fatty acid levels to investigate further metabolic effects of protein supplementation in the older adults. Future studies might include such outcomes to gain more insight into the mechanisms behind the relationship between protein supplementation, metabolism, appetite regulation, and microbiota composition.

## 5. Conclusions

We have previously shown that dietary advice to increase protein intake to ≥1.2 g/kg aBW/day improved physical functioning [21]. Here, we demonstrate that this advice did not significantly affect appetite, CNS responses to food cues, or the gut microbiota. Therefore, raising the RDA for protein intake in older adults can be done without concerns of negatively impacting the gut microbiota or suppressing appetite.

## Figures and Tables

**Table 1:** Baseline characteristics of microbiota (*n* = 90) and fMRI sub-samples (*n* = 48).

	Microbiota Sample			fMRI Subsample		
	Diet Group (*n* = 47)	Control Group (*n* = 43)	*p*-Value	Diet Group (*n* = 27)	Control Group (*n* = 21)	*p*-Value
**Demographics**						
Age (years)	74.6 ± 4.8	74.1 ± 4.7	0.572	73 (70–80)	72 (71–75)	0.445
Sex (male)	28 (59.6)	19 (44.2)	0.205	18 (66.7)	12 (57.1)	0.558
BMI (kg/m^2^)	26.1 ± 2.9	26.8 ± 2.9	0.227	26.6 ± 2.7	27.1 ± 2.9	0.604
MMSE	29 (27–30)	29 (27–30)	0.573	29 (27–29)	29 (27–30)	0.734
Education			0.110			0.565
Low	3 (6.4)	0 (0.0)		2 (7.4)	0 (0.0)	
Middle	8 (17.0)	13 (30.2)		5 (18.5)	6 (28.6)	
High	36 (76.6)	30 (69.8)		20 (74.1)	15 (71.4)	
Study site (Amsterdam)	35 (74.5)	33 (76.7)	1.000			
**Appetite**						
SNAQ appetite score	14.9 ± 1.7	15.0 ± 2.1	0.965	15.2 ± 1.4	15.2 ± 2.4	0.978
VAS appetite scores				4.7 (3.3–5.8)	5.3 (3.3–6.4)	0.560
**Food intake**						
Energy intake (kcal/day)	1701.9 ± 427.4	1611.0 ± 301.8	0.244	1740.4 ± 464.5	1690.2 ± 311.1	0.657
Protein intake (g/kg aBW/day)	0.8 ± 0.2	0.8 ± 0.1	0.689	0.8 ± 0.2	0.8 ± 0.1	0.777
Protein intake (g/day)	62.9 ± 14.0	60.9 ± 10.4	0.457	64.5 ± 14.6	62.0 ± 11.2	0.526
Carbohydrate intake (g/day)	181.4 ± 56.4	170.9 ± 47.6	0.347	182.5 ± 63.2	182.2 ± 49.3	0.986
Fat intake (g/day)	67.3 ± 20.4	66.1 ± 18.3	0.771	69.2 ± 21.9	69.8 ± 20.1	0.929

BMI: body mass index; MMSE: Mini-Mental State Exam; SNAQ: Simplified Nutritional Appetite Questionnaire; VAS: Visual Analogue Scale; aBW: adjusted body weight; fMRI: functional magnetic resonance imaging. Values are depicted in mean ± standard deviation (parametric continuous), median (interquartile range) (non-parametric continuous), or number (%) (categorical). Baseline differences between groups are tested by independent samples *t*-test (parametric continuous), independent samples Mann–Whitney U Test (non-parametric continuous), or Fisher’s exact test (categorical).

**Table 2:** Dietary intake after 3 and 6 months in the intervention and control groups.

**Microbiota Subsample**	**Diet Group (*n* = 47)**		**Control Group (*n* = 43)**		**Between-Group Differences**	
	**3 Months**	**6 Months**	**3 Months**	**6 Months**	**B (95%-CI)**	***p*-Value**
Energy intake (kcal/day)	1873.9 ± 454.2	1836.1 ± 382.9	1679.2 ± 429.8	1699.2 ± 341.6	104.0 (0.4–207.5)	0.0008 *
Protein intake (g/kg aBW/day	1.3 ± 0.3	1.2 ± 0.2	0.9 ± 0.2	0.9 ± 0.2	0.4 (0.3–0.4)	<0.0001 *
Protein intake (g/day)	96.2 ± 26.7	94.2 ± 22.2	64.1 ± 15.0	66.8 ± 15.2	27.1 (21.2–33.0)	<0.0001 *
Carbohydrate intake (g/day)	187.7 ± 51.0	185.0 ± 48.9	177.0 ± 54.6	174.2 ± 47.7	4.2 (−7.9–16.3)	0.0367 *
Fat intake (g/day)	72.4 ± 24.4	69.9 ± 23.0	68.6 ± 24.9	71.5 ± 18.3	0.2 (−6.3–6.6)	0.6514
**fMRI Subsample**	**Diet Group (*n* = 27)**		**Control Group (*n* = 21)**		**Between-Group Differences**	
	**3 Months**	**6 Months**	**3 Months**	**6 Months**	**B (95%-CI)**	***p*-Value**
Energy intake (kcal/day)	1893.3 ± 484.3	1847.4 ± 402.8	1815.0 ± 470.2	1764.1 ± 310.7	70.4 (−86.3–227.1)	0.1897
Protein intake (g/kg aBW/day	1.3 ± 0.3	1.3 ± 0.2	0.9 ± 0.2	0.9 ± 0.2	0.4 (0.3–0.5)	<0.0001 *
Protein intake (g/day)	100.8 ± 26.6	101.3 ± 19.8	66.4 ± 14.4	67.9 ± 15.6	33.1 (24.5–41.7)	<0.0001 *
Carbohydrate intake (g/day)	187.6 ± 52.5	180.9 ± 54.0	194.3 ± 61.3	183.2 ± 46.6	−2.5 (−20.8–15.8)	0.8486
Fat intake (g/day)	72.7 ± 27.1	69.9 ± 25.8	74.2 ± 25.1	74.3 ± 15.8	−1.8 (−11.5–8.0)	0.7255

aBW: adjusted body weight; 95%-CI: 95% confidence interval; B: regression coefficient. Shown are means ± standard deviations and regression coefficients (95%-confidence intervals). Differences between groups in food intake were linear mixed models with random intercept for the participant, adjusted for baseline values. * *p*-value < 0.05.

**Table 3:** Appetite measures at baseline and after 6 months in the intervention and control groups.

	Diet Group		Control Group		Difference at 6 Months	
	Baseline	6 Months	Baseline	6 Months	B (95%-CI)	*p*-Value
SNAQ appetite score (*n* = 90)	14.9 ± 1.7	14.9 ± 2.1	15.0 ± 2.1	15.3 ± 2.1	−0.36 (−1.10–0.39)	0.347
Average VAS appetite score (*n* = 48)	4.7 (3.3–5.8)	4.5 (2.8–6.3)	5.3 (3.3–6.4)	5.0 (3.8–6.2)	−0.20 (−1.10–0.70)	0.657
How hungry are you	4.0 (2.0–6.0)	3.0 (1.0–6.0)	4.1 (1.0–6.0)	4.5 (1.7–6.0)	−0.62 (−1.80–0.56)	0.293
How full are you	2.9 (2.0–5.0)	2.0 (1.0–3.5)	2.6 (1.0–5.4)	3.0 (2.0–5.5)	−1.06 (−2.35–0.23)	0.104
How eager are you to eat	5.0 (3.0–7.0)	5.0 (2.0–6.0)	6.8 (2.0–7.1)	5.9 (4.2–7.0)	−0.44 (−1.65–0.77)	0.469
How much could you eat	5.0 (3.9–6.9)	5.0 (3.0–5.5)	6.0 (4.0–7.0)	5.5 (4.6–7.0)	−0.77 (−1.80–0.26)	0.139
How strong is the urge to eat	4.0 (2.0–6.0)	4.0 (2.0–6.0)	5.0 (0.8–7.0)	5.0 (2.0–6.0)	−0.15 (−1.50–1.20)	0.825
How strong is the thought of food in your head	3.0 (1.5–7.0)	3.0 (1.0–6.0)	5.0 (1.0–7.0)	5.0 (2.0–6.0)	−0.48 (−1.93–0.97)	0.507

SNAQ: Simplified Nutrition Appetite Questionnaire; VAS: Visual Analogue Scale. Shown are means ± standard deviations, medians (interquartile ranges), and regression coefficients (95%-confidence intervals). Differences between groups in appetite were tested with linear regression for follow-up values, adjusted for baseline values.

**Table 4:** Significant results of the main effects observed in the fMRI virtual food task of whole-brain analyses.

	Side				MNI			
		Cluster	Voxels	Z-Max	x	y	z	*p*-Value *
**Food vs. non-food pictures**								
Lateral occipital cortex Lateral occipital cortex Superior parietal lobe Cingulate gyrus Superior parietal lobe Lateral occipital cortex	R L R L L L	6 5 4 3 2 1	2751 2418 478 205 201 201	7.01 6.52 5.5 4.59 4.19 4.05	40 −44 32 −6 −34 −26	−78 −74 −52 −54 −54 −64	−10 −4 62 26 66 30	2.15 × 10^−17^ 6.3 × 10^−16^ 3.92 × 10^−5^ 0.0096 0.0106 0.0106
**High-caloric vs. non-food pictures**								
Insula	L	7	218	5.73	−44	−30	34	0.00914
Lateral occipital cortex Lateral occipital cortex Lateral occipital cortex Cingulate gyrus Superior parietal lobe Supramarginal gyrus	R L L L L L	6 5 4 3 2 1	4150 3185 295 289 206 156	7.08 6.77 4.52 4.72 4.46 4.61	46 −44 −20 −6 −32 −44	−72 −74 −82 −54 −56 −30	0 −4 44 26 66 34	4.75 × 10^−22^ 2.02 × 10^−18^ 0.00182 0.00205 0.0119 0.0383

Depicted are the main effects of the two contrasts (food versus non-food and high-caloric versus non-food images) of the virtual fMRI paradigm. To this end, fMRI baseline data of all participants across both groups was entered into a one-sample *t*-test and assessed on whole-brain level with a threshold *p* < 0.05. * *p*-values are corrected for multiple comparisons using family-wise error (FWE). Cluster information was obtained using the ‘cluster’ tool from FSL; *p*-values depict significance of clusters using Gaussian random field (GRF) theory. Montreal Neurological Institute (MNI): anatomical coordinates of peak voxels activated per separate brain region. Z-max: maximum z-score within the identified cluster; L: left; R: right.

**Table 5:** Effect of intervention on responses to fMRI virtual and palatable food tasks in pre-defined regions of interest.

	Picture Task				Palatable Food Task			
	Food vs. Non-Food		High Caloric vs. Non-Food		Anticipation Chocolate vs. Tasteless Solution		Receipt Chocolate vs. Tasteless Solution	
	B (95%-CI)	*p*-Value	B (95%-CI)	*p*-Value	B (95%-CI)	*p*-Value	B (95%-CI)	*p*-Value
Putamen right	−0.02 (−0.08–0.03)	0.385	−0.04 (−0.11–0.03)	0.255	0.03 (−0.05–0.11)	0.485	−0.02 (−0.07–0.03)	0.495
Insula right	−0.01 (−0.07–0.05)	0.750	−0.04 (−0.11–0.03)	0.272	0.07 (0.00–0.13)	0.045 *	0.01 (−0.03–0.06)	0.474
Amygdala right	−0.06 (−0.15–0.03)	0.178	−0.07 (−0.19–0.05)	0.260	0.03 (−0.08–0.13)	0.614	−0.03 (−0.12–0.06)	0.475
Caudate nucleus right	−0.02 (−0.07–0.03)	0.394	−0.04 (−0.11–0.04)	0.307	0.06 (−0.01–0.14)	0.091	−0.03 (−0.09–0.02)	0.220
Putamen left	−0.04 (−0.09–0.01)	0.151	−0.05 (−0.12–0.02)	0.157	0.03 (−0.03–0.10)	0.334	−0.04 (−0.10–0.01)	0.134
Insula left	−0.04 (−0.10–0.03)	0.256	−0.05 (−0.12–0.02)	0.132	0.03 (−0.04–0.11)	0.385	−0.01 (−0.05–0.04)	0.759
Amygdala left	−0.04 (−0.13–0.06)	0.445	−0.07 (−0.19–0.05)	0.227	0.09 (−0.01–0.19)	0.063	−0.05 (−0.14–0.03)	0.224
Caudate nucleus left	−0.02 (−0.07–0.04)	0.480	−0.03 (−0.11–0.04)	0.359	0.04 (−0.04–0.11)	0.305	−0.04 (−0.09–0.02)	0.162

Shown are regression coefficients (95%-confidence intervals) and *p*-values. Percentual differences between groups in blood-oxygen level dependent signal were tested with linear regression for follow-up values, adjusted for baseline values. CI: confidence interval; B: regression coefficient. * *p*-value < 0.05.

## Data Availability

This clinical trial data can be requested by any qualified researcher who engage in rigorous, independent scientific research, and will be provided upon reasonable request by the corresponding author.

## References

[1] Eide H.K., Benth J.Š, Sortland K., Halvorsen K., Almendingen K. (2015). Prevalence of nutritional risk in the non-demented hospitalised elderly: A cross-sectional study from Norway using stratified sampling. J. Nutr. Sci..

[2] Almohaisen N., Gittins M., Todd C., Sremanakova J., Sowerbutts A.M., Aldossari A., Almutairi A., Jones D., Burden S. (2022). Prevalence of Undernutrition, Frailty and Sarcopenia in Community-Dwelling People Aged 50 Years and Above: Systematic Review and Meta-Analysis. Nutrients.

[3] Deutz N.E., Bauer J.M., Barazzoni R., Biolo G., Boirie Y., Bosy-Westphal A., Cederholm T., Cruz-Jentoft A., Krznariç Z., Nair K.S. (2014). Protein intake and exercise for optimal muscle function with aging: Recommendations from the ESPEN Expert Group. Clin. Nutr..

[4] Kim D., Park Y. (2020). Amount of Protein Required to Improve Muscle Mass in Older Adults. Nutrients.

[5] EFSA (2012). Scientific Opinion on Dietary Reference Values for protein. EFSA Panel on Dietetic Products, Nutrition and Allergies (NDA). EFSA J..

[6] Ouyang Y., Huang F., Zhang X., Li L., Zhang B., Wang Z., Wang H. (2022). Association of Dietary Protein Intake with Muscle Mass in Elderly Chinese: A Cross-Sectional Study. Nutrients.

[7] Morgan P.T., Harris D.O., Marshall R.N., Quinlan J.I., Edwards S.J., Allen S.L., Breen L. (2021). Protein Source and Quality for Skeletal Muscle Anabolism in Young and Older Adults: A Systematic Review and Meta-Analysis. J. Nutr..

[8] Lonnie M., Hooker E., Brunstrom J.M., Corfe B.M., Green M.A., Watson A.W., Williams E.A., Stevenson E.J., Penson S., Johnstone A.M. (2018). Protein for Life: Review of Optimal Protein Intake, Sustainable Dietary Sources and the Effect on Appetite in Ageing Adults. Nutrients.

[9] Paddon-Jones D., Westman E., Mattes R.D., Wolfe R.R., Astrup A., Westerterp-Plantenga M. (2008). Protein, weight management, and satiety. Am. J. Clin. Nutr..

[10] Lejeune M.P.G.M., Westerterp K.R., Adam T.C.M., Luscombe-Marsh N., Westerterp-Plantenga M.S. (2006). Ghrelin and glucagon-like peptide 1 concentrations, 24-h satiety, and energy and substrate metabolism during a high-protein diet and measured in a respiration chamber. Am. J. Clin. Nutr..

[11] Soenen S., Giezenaar C., Hutchison A.T., Horowitz M., Chapman I., Luscombe-Marsh N. (2014). Effects of intraduodenal protein on appetite, energy intake, and antropyloroduodenal motility in healthy older compared with young men in a randomized trial. Am. J. Clin. Nutr..

[12] van der Pols-Vijlbrief R., Wijnhoven H.A., Schaap L.A., Terwee C.B., Visser M. (2014). Determinants of protein-energy malnutrition in community-dwelling older adults: A systematic review of observational studies. Ageing Res. Rev..

[13] Jahan-Mihan A., Luhovyy B.L., El Khoury D., Anderson G.H. (2011). Dietary Proteins as Determinants of Metabolic and Physiologic Functions of the Gastrointestinal Tract. Nutrients.

[14] Drummen M., Dorenbos E., Vreugdenhil A.C.E., Stratton G., Raben A., Westerterp-Plantenga M.S., Adam T.C. (2018). Associations of Brain Reactivity to Food Cues with Weight Loss, Protein Intake and Dietary Restraint during the PREVIEW Intervention. Nutrients.

[15] Leidy H.J., Ortinau L.C., Douglas S.M., Hoertel H.A. (2013). Beneficial effects of a higher-protein breakfast on the appetitive, hormonal, and neural signals controlling energy intake regulation in overweight/obese, “breakfast-skipping,” late-adolescent girls. Am. J. Clin. Nutr..

[16] Griffioen-Roose S., Smeets P.A., van den Heuvel E., Boesveldt S., Finlayson G., De Graaf C. (2014). Human protein status modulates brain reward responses to food cues. Am. J. Clin. Nutr..

[17] Russell W.R., Gratz S.W., Duncan S.H., Holtrop G., Ince J., Scobbie L., Duncan G., Johnstone A.M., Lobley G.E., Wallace R.J. (2011). High-protein, reduced-carbohydrate weight-loss diets promote metabolite profiles likely to be detrimental to colonic health. Am. J. Clin. Nutr..

[18] Fluitman K.S., De Clercq N.C., Keijser B.J., Visser M., Nieuwdorp M., Ijzerman R.G. (2017). The intestinal microbiota, energy balance, and malnutrition: Emphasis on the role of short-chain fatty acids. Expert Rev. Endocrinol. Metab..

[19] Strasser B., Wolters M., Weyh C., Krüger K., Ticinesi A. (2021). The Effects of Lifestyle and Diet on Gut Microbiota Composition, Inflammation and Muscle Performance in Our Aging Society. Nutrients.

[20] Reinders I., Wijnhoven H.A.H., Jyväkorpi S.K., Suominen M.H., Niskanen R., Bosmans J.E., Brouwer I.A., Fluitman K.S., Klein M.C.A., Kuijper L.D. (2020). Effectiveness and cost-effectiveness of personalised dietary advice aiming at increasing protein intake on physical functioning in community-dwelling older adults with lower habitual protein intake: Rationale and design of the PROMISS randomised controlled trial. BMJ Open.

[21] Reinders I., Visser M., Jyväkorpi S.K., Niskanen R.T., Bosmans J.E., Ângela J.B., Ingeborg A.B., Lothar D.K., Margreet R.O., Kaisu H.P. (2022). The cost effectiveness of personalized dietary advice to increase protein intake in older adults with lower habitual protein intake: A randomized controlled trial. Eur. J. Nutrition.

[22] Folstein M.F., Folstein S.E., McHugh P.R. (1975). “Mini-Mental State”. A Practical Method for Grading the Cognitive State of Patients for the Clinician. J. Psychiatr. Res..

[23] Grasso A.C., Olthof M.R., Reinders I., Wijnhoven H.A.H., Visser M., Brouwer I.A. (2022). Effect of personalized dietary advice to increase protein intake on food consumption and the environmental impact of the diet in community-dwelling older adults: Results from the PROMISS trial. Eur. J. Nutrition.

[24] Wilson M.-M.G., Thomas D.R., Rubenstein L., Chibnall J.T., Anderson S., Baxi A., Diebold M.R., Morley J.E. (2005). Appetite assessment: Simple appetite questionnaire predicts weight loss in community-dwelling adults and nursing home residents. Am. J. Clin. Nutr..

[25] Salonen A., Nikkilä J., Jalanka-Tuovinen J., Immonen O., Rajilić-Stojanović M., Kekkonen R.A., Palva A., de Vos W.M. (2010). Comparative analysis of fecal DNA extraction methods with phylogenetic microarray: Effective recovery of bacterial and archaeal DNA using mechanical cell lysis. J. Microbiol. Methods.

[26] Kozich J.J., Westcott S.L., Baxter N.T., Highlander S.K., Schloss P.D. (2013). Development of a dual-index sequencing strategy and curation pipeline for analyzing amplicon sequence data on the MiSeq Illumina sequencing platform. Appl. Environ. Microbiol..

[27] Edgar R.C. (2010). Search and clustering orders of magnitude faster than BLAST. Bioinformatics.

[28] Edgar R.C. (2016). UNOISE2: Improved error-correction for Illumina 16S and ITS amplicon sequencing. BioRxiv..

[29] Wang Q., Garrity G.M., Tiedje J.M., Cole J.R. (2007). Naive Bayesian classifier for rapid assignment of rRNA sequences into the new bacterial taxonomy. Appl. Env. Microbiol..

[30] Quast C., Pruesse E., Yilmaz P., Gerken J., Schweer T., Yarza P., Peplies J., Glöckner F.O. (2013). The SILVA ribosomal RNA gene database project: Improved data processing and web-based tools. Nucleic Acids Res..

[31] van Bloemendaal L., IJzerman R.G., Kulve J.S.T., Barkhof F., Konrad R.J., Drent M.L., Veltman D.J., Diamant M. (2014). GLP-1 receptor activation modulates appetite- and reward-related brain areas in humans. Diabetes.

[32] Esteban O., Markiewicz C.J., Blair R.W., Moodie C.A., Isik A.I., Erramuzpe A., Kent J.D., Goncalves M., DuPre E., Snyder M. (2018). fMRIPrep: A robust preprocessing pipeline for functional MRI. Nat. Methods.

[33] Esteban O., Ciric R., Finc K., Blair R.W., Markiewicz C.J., Moodie C.A., Kent J.D., Goncalves M., DuPre E., Gomez D.E.P. (2020). Analysis of task-based functional MRI data preprocessed with fMRIPrep. Nat. Protoc..

[34] Fonov V., Evans A., McKinstry R., Almli C., Collins D. (2009). Unbiased nonlinear average age-appropriate brain templates from birth to adulthood. Neuroimage.

[35] Jenkinson M., Bannister P., Brady M., Smith S. (2002). Improved Optimization for the Robust and Accurate Linear Registration and Motion Correction of Brain Images. Neuroimage.

[36] Cox R.W., Hyde J.S. (1997). Software tools for analysis and visualization of fMRI data. NMR Biomed..

[37] Jenkinson M., Smith S. (2001). A global optimisation method for robust affine registration of brain images. Med. Image Anal..

[38] Pruim R.H.R., Mennes M., van Rooij D., Llera A., Buitelaar J.K., Beckmann C.F. (2015). ICA-AROMA: A robust ICA-based strategy for removing motion artifacts from fMRI data. NeuroImage.

[39] van Bloemendaal L., Veltman D.J., Kulve J.S.T., Groot P.F., Ruhe H.G., Barkhof F., Diamant M., Ijzerman R.G. (2015). Brain reward-system activation in response to anticipation and consumption of palatable food is altered by glucagon-like peptide-1 receptor activation in humans. Diabetes Obes. Metab..

[40] Stice E., Spoor S., Bohon C., Small D.M. (2008). Relation between obesity and blunted striatal response to food is moderated by TaqIA A1 allele. Science.

[41] Moreno-Pérez D., Bressa C., Bailén M., Hamed-Bousdar S., Naclerio F., Carmona M., Pérez M., González-Soltero R., Montalvo-Lominchar M.G., Carabaña C. (2018). Effect of a Protein Supplement on the Gut Microbiota of Endurance Athletes: A Randomized, Controlled, Double-Blind Pilot Study. Nutrients.

[42] Schaafsma A., Mallee L., Belt M.V.D., Floris E., Kortman G., Veldman J., Ende D.V.D., Kardinaal A. (2021). The Effect of A Whey-Protein and Galacto-Oligosaccharides Based Product on Parameters of Sleep Quality, Stress, and Gut Microbiota in Apparently Healthy Adults with Moderate Sleep Disturbances: A Randomized Controlled Cross-Over Study. Nutrients.

[43] Doornweerd S., De Geus E.J., Barkhof F., Van Bloemendaal L., Boomsma D.I., Van Dongen J., Drent M.L., Willemsen G., Veltman D.J., Ijzerman R.G. (2017). Brain reward responses to food stimuli among female monozygotic twins discordant for BMI. Brain Imaging Behav..

[44] van Ruiten C.C., Veltman D.J., Wijdeveld M., Kulve J.S.T., Kramer M.H.H., Nieuwdorp M., IJzerman R.G. (2022). Combination therapy with exenatide decreases the dapagliflozin-induced changes in brain responses to anticipation and consumption of palatable food in patients with type 2 diabetes: A randomized controlled trial. Diabetes Obes. Metabolism..

[45] Kulve J.S.T., Veltman D.J., van Bloemendaal L., Barkhof F., Drent M.L., Diamant M., IJzerman R.G. (2016). Liraglutide Reduces CNS Activation in Response to Visual Food Cues Only After Short-term Treatment in Patients With Type 2 Diabetes. Diabetes Care..

[46] Smith S.M., Jenkinson M., Woolrich M.W., Beckmann C.F., Behrens T.E., Johansen-Berg H., Bannister P.R., De Luca M., Drobnjak I., Flitney D.E. (2004). Advances in functional and structural MR image analysis and implementation as FSL. Neuroimage.

[47] Born J.M., Lemmens S.G.T., Rutters F., Nieuwenhuizen A., Formisano E., Goebel R., Westerterp-Plantenga M.S. (2009). Acute stress and food-related reward activation in the brain during food choice during eating in the absence of hunger. Int. J. Obes..

[48] Farr O.M., Li C.-S.R., Mantzoros C.S. (2016). Central nervous system regulation of eating: Insights from human brain imaging. Metabolism.

[49] Wright H., Li X., Fallon N.B., Crookall R., Giesbrecht T., Thomas A., Halford J.C., Harrold J., Stancak A. (2016). Differential effects of hunger and satiety on insular cortex and hypothalamic functional connectivity. Eur. J. Neurosci..

[50] Pursey K.M., Stanwell P., Callister R.J., Brain K., Collins C.E., Burrows T.L. (2014). Neural Responses to Visual Food Cues According to Weight Status: A Systematic Review of Functional Magnetic Resonance Imaging Studies. Front. Nutr..

[51] Team R.C. (2018). R: A Language and Environment for Statistical Computing. http://www.R-project.org/.

[52] McMurdie P.J., Holmes S. (2013). phyloseq: An R package for reproducible interactive analysis and graphics of microbiome census data. PLoS ONE.

[53] Oksanen J., Blanchet F.G., Kindt R., Legendre P., O’hara R.B., Simpson G.L., Solymos P., Stevens M.H.H., Wagner H. (2018). Vegan: Community Ecology Package. https://CRAN.R-project.org/package=vegan.

[54] Wickham H. (2016). Elegant Graphics for Data Analysis.

[55] Love M.I., Huber W., Anders S. (2014). Moderated estimation of fold change and dispersion for RNA-seq data with DESeq2. Genome Biol..

[56] Hoffman G.E., Schadt E.E. (2016). variancePartition: Interpreting drivers of variation in complex gene expression studies. BMC Bioinform..

[57] Hoffman G.E., Roussos P. (2021). Dream: Powerful differential expression analysis for repeated measures designs. Bioinformatics.

[58] Benjamini Y., Hochberg Y. (1995). Controlling the False Discovery Rate: A Practical and Powerful Approach to Multiple Testing. J. R. Stat. Soc. Ser. B.

[59] Salem V., Dhillo W.S. (2015). IMAGING IN ENDOCRINOLOGY: The use of functional MRI to study the endocrinology of appetite. Eur. J. Endocrinol..

[60] Brooks S.J., Cedernaes J., Schiöth H.B. (2013). Increased Prefrontal and Parahippocampal Activation with Reduced Dorsolateral Prefrontal and Insular Cortex Activation to Food Images in Obesity: A Meta-Analysis of fMRI Studies. PLoS ONE.

[61] Boswell R.G., Kober H. (2016). Food cue reactivity and craving predict eating and weight gain: A meta-analytic review. Obes. Rev..

[62] Giezenaar C., Trahair L.G., Rigda R., Hutchison A.T., Feinle-Bisset C., Luscombe-Marsh N.D., Hausken T., Jones K.L., Horowitz M., Chapman I. (2015). Lesser suppression of energy intake by orally ingested whey protein in healthy older men compared with young controls. Am. J. Physiol. Integr. Comp. Physiol..

[63] David L.A., Maurice C.F., Carmody R.N., Gootenberg D.B., Button J.E., Wolfe B.E., Ling A.V., Devlin A.S., Varma Y., Fischbach M.A. (2014). Diet rapidly and reproducibly alters the human gut microbiome. Nature.

[64] Ma N., Tian Y., Wu Y., Ma X. (2017). Contributions of the Interaction Between Dietary Protein and Gut Microbiota to Intestinal Health. Curr. Protein Pept. Sci..

[65] Bauer J., Biolo G., Cederholm T., Cesari M., Cruz-Jentoft A.J., Morley J.E., Phillips S., Sieber C., Stehle P., Teta D. (2013). Evidence-Based Recommendations for Optimal Dietary Protein Intake in Older People: A Position Paper From the PROT-AGE Study Group. J. Am. Med. Dir. Assoc..

[66] Ben-Harchache S., Roche H.M., Corish C.A., Horner K.M. (2021). The Impact of Protein Supplementation on Appetite and Energy Intake in Healthy Older Adults: A Systematic Review with Meta-Analysis. Adv. Nutr. Int. Rev. J..

[67] Rogers H.L., Fernandez S.N., Hernando S.P., Sanchez A., Martos C., Moreno M., Grandes G. (2021). “My Patients Asked Me If I Owned a Fruit Stand in Town or Something.” Barriers and Facilitators of Personalized Dietary Advice Implemented in a Primary Care Setting. J. Pers. Medicine.

